# Effects of hypoxia and hyperoxia on exercise-induced metabolomic and transcriptomic profiles in equine skeletal muscle

**DOI:** 10.1242/jeb.250956

**Published:** 2025-12-17

**Authors:** Kenya Takahashi, Kazutaka Mukai, Yuji Takahashi, Yusaku Ebisuda, Fumi Sugiyama, Hideo Hatta, Yu Kitaoka

**Affiliations:** ^1^ Department of Sports Sciences, The University of Tokyo, Tokyo 153-8902, Japan; ^2^ Sports Science Division, Equine Research Institute, Japan Racing Association, Tochigi 329-0412, Japan; ^3^ Department of Human Sciences, Kanagawa University, Kanagawa 221-8686, Japan

**Keywords:** Exercise, Metabolomics, Oxygen, RNA sequencing, Thoroughbred

## Abstract

To explore the molecular mechanisms underlying oxygen-dependent regulation of skeletal muscle adaptations, eight Thoroughbred horses performed 2 min of exercise at a velocity corresponding to 95% maximal O_2_ uptake under a normoxic condition, while using inspired O_2_ levels of 0.21 (normoxia), 0.26 (hyperoxia) or 0.16 (hypoxia). At the end of the exercise, arterial O_2_ saturation was significantly higher with hyperoxia and lower with hypoxia than with normoxia. However, no significant difference in plasma lactate or muscle glycogen concentrations was observed across the O_2_ conditions. A metabolomic analysis showed that muscle metabolite concentrations involved in glycolysis and the tricarboxylic acid cycle significantly changed in response to exercise but did not significantly differ across the O_2_ conditions. RNA-sequencing data showed that fewer genes were significantly altered by acute exercise in hyperoxia (upregulated: 523; downregulated: 116) and hypoxia (upregulated: 857; downregulated: 320) compared with normoxia (upregulated: 1628, downregulated: 924). Among them, numerous genes, including well-known exercise-responsive genes, such as *NR4A3*, *PPARGC1A*, *PDK4* and *VEGFA*, were altered following exercise, irrespective of the O_2_ environment. Hyperoxic exercise induced responses of genes related to lysosomal activity, such as *M6PR* and *CTNS*, whereas hypoxic exercise triggered hypoxia-responsive gene expression, including *PIK3R1*, *THPO* and *AKAP1*. These findings suggest that arterial O_2_ availability does not necessarily alter global metabolic or transcriptomic response following a single exercise bout in horses. However, inspired O_2_ fraction-specific gene responses may play roles in long-term skeletal muscle adaptations and could contribute to the development of optimized training strategies for improved well-being and performance.

## Teaser

**Summary:** Although altered arterial O_2_ concentrations have little effect on exercise-induced changes in metabolites, transcriptomic analysis reveals genes that are specific to hyperoxic and hypoxic exercise.

## INTRODUCTION

Athletic performance is supported by the supply of oxygen (O_2_) to working muscles. It is well established that exercise performance and O_2_ uptake are reduced in the hypoxic environment, whereas inspiring a hyperoxic gas during exercise may improve performance, leading to higher and/or longer power output (Ulrich et al., 2017). Varying the fraction of inspired O_2_ (*F*i_O_2__) has been used in an attempt to increase training benefits by altering metabolic or mechanical stress during exercise (Cardinale and Ekblom, 2018; Millet et al., 2016). Although exercise-induced systemic physiological markers (e.g. cytokines and hormones) are reported to be affected by environmental stressors such as *F*i_O_2__ (Burtscher et al., 2024), the underlying molecular mechanisms of skeletal muscle adaptation remain unclear.

Thoroughbred horses are highly athletic, and their body-mass-specific maximal O_2_ uptake can reach twice as high as that of elite athletes (Kitaoka et al., 2011; Poole and Erickson, 2011). Unlike humans, in which hypoxemia can occur in certain populations, especially in fit athletes (Dempsey and Wagner, 1999; Harms et al., 2000; Powers et al., 1989), horses universally experience exercise-induced hypoxemia (Art and Lekeux, 1995; Erickson et al., 1994; Wagner et al., 1989). Our research group and others have previously reported that this exercise-induced hypoxemia in Thoroughbred horses is exacerbated by hypoxia, but relieved by hyperoxia (Ohmura et al., 2020; Wagner et al., 1996). These traits make Thoroughbred horses an ideal model for investigating the effects of varied *F*i_O_2__ levels on metabolic and transcriptional responses to exercise.

This study aimed to provide a comprehensive understanding of the potential role of O_2_ in the regulation of skeletal muscle adaptations to exercise in Thoroughbred horses. In this study, we first demonstrated that neither acute hyperoxia nor hypoxia had a significant effect on metabolic responses to a 2-min exercise bout at 95% maximal oxygen consumption (*V̇*_O_2_,max_), despite varying arterial O_2_ availability. We performed a metabolomic analysis and RNA sequencing (RNA-seq), which are powerful and unbiased approaches that uncover global responses, to determine whether exercise-induced transcriptional responses are O_2_ dependent in skeletal muscle.

## MATERIALS AND METHODS

### Animals and ethical approval

Eight Thoroughbred horses were used in this study (Table 1). The experimental protocols for the study were reviewed and approved by the Animal Welfare and Ethics Committee of the Japan Racing Association (JRA) Equine Research Institute (approval number: 24-7). The horses had a carotid artery surgically moved from the carotid sheath to a subcutaneous location under sevoflurane anesthesia to facilitate arterial catheterization. All incisions for catheter placement and muscle biopsies were performed under local anesthesia using lidocaine. The horses had participated in our previous studies and were well acclimated to the experimental procedures used in the present study (Kitaoka et al., 2020; Mukai et al., 2021; Takahashi et al., 2025; Wang et al., 2020). All efforts were made to minimize discomfort of the horses.

**Table 1.** Animal characteristics and running speed

Variable	Mean±s.d. (range)
Age (years)	6±2 (3–9)
Sex	5 females and 3 castrated males
Body mass (kg)	510±33 (470–583)
Maximal O_2_ uptake (ml min^−1^ kg^−1^)	144±20 (118–170)
Running speed during exercise bout (m s^−1^)	10.4±0.4 (9.8–11.1)
	

Values are means±s.d.; *n*=8.

### Preliminary incremental exercise test

The horses performed incremental exercise tests on a treadmill 1 week before the first session of high-intensity interval exercise to determine the speed that elicited the maximal O_2_ consumption of each horse. Following a 3-min warm-up at 4 m s^−1^, the horses exercised for 2 min each at 1.7, 4, 6, 8, 10, 12 and 13 m s^−1^ on a treadmill with a 6% incline until they could no longer maintain their position with human encouragement. Horses wore a 25-cm diameter open-flow mask on the treadmill through which a rheostat-controlled blower drew air. Air flowed through 25-cm diameter tubing and across a pneumotachograph (LF-150B, Vise Medical, Chiba, Japan) connected to a differential pressure transducer (TF-5, Vise Medical). O_2_ and carbon dioxide concentrations were measured with an analyzer (FC-10 oxygen analyzer and CA-10 carbon dioxide analyzer, Sable Systems International, Las Vegas, NV, USA), and calibrations were performed using electronic mass flow meters (CR-300, Kofloc, Kyoto, Japan) with the nitrogen-dilution/carbon dioxide-addition mass-balance technique. Gas analyzer and mass flowmeter outputs for the last 30 s of each step were recorded on personal computers using commercial hardware and software (DI-720 and Windaq Pro+, DATAQ, Akron, OH, USA) with sampling at 200 Hz.

### Experimental design

**Fig. 1.**
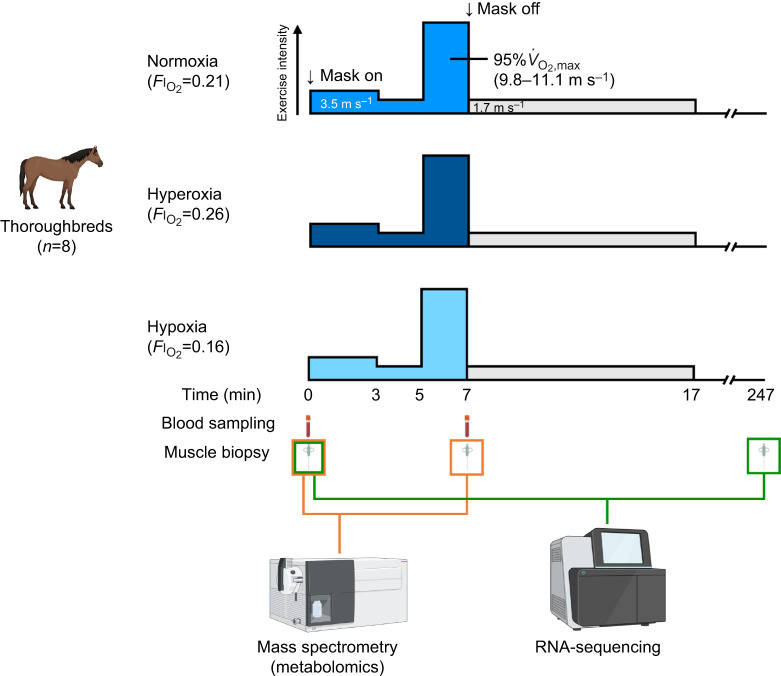
Schematics of the experimental design.

A schematic of the experimental design is shown in Fig. 1. In a randomized, crossover design, eight Thoroughbred horses exercised under normoxia, hyperoxia or hypoxia, separated by a 7-day washout period, during which the horses were pastured in a yard and walked for 1 h day^−1^ in a walker without treadmill exercise. Before the first exercise session, body mass was measured using a weight scale (RT-1C, Kubota, Osaka, Japan). In each session, catheters were placed and a heart rate monitor (S810, Polar, Kempele, Finland) was attached. Resting muscle samples from the gluteus medius muscle and arterial and mixed venous blood samples were obtained before exercise. After the horses wore the open-flow mask to inspire designated O_2_ concentrations, they warmed up with a 3-min trot at 3.5 m s^−1^ and a 2-min walk at 1.7 m s^−1^. The horses then performed a 2-min exercise bout at the speed that elicited 95% of their maximal O_2_ consumption measured under normoxia. This exercise intensity and duration were selected based on the protocol used in our previous study, where hypoxic training resulted in greater performance improvements compared with normoxic training (Wang et al., 2020). At the end of the exercise, the mask was removed, and samples were collected again, including biopsies from the gluteus medius muscle and arterial and mixed venous blood from the carotid and pulmonary arteries, respectively. The horses then cooled down at 1.7 m s^−1^ for 10 min. At 4 h after each exercise session, additional biopsies were collected from the gluteus medius.

### Hyperoxic and hypoxic gas supply

The N₂-dilution calibration technique for the open-flow system was performed as previously described (Fedak et al., 1981), and the procedure of hyperoxic and hypoxic gas supply was slightly modified from the method previously described (Mukai et al., 2020). Briefly, a mixing chamber was connected to the upstream flexible tube on the open-flow mask through which a flow of nitrogen and O_2_ was blown into the upstream end of the flow system and mixed with a bias flow of air of 80–120 l s^−1^ to create the designated inspired O_2_ concentration. Nitrogen and O_2_ gas flow was controlled with a mass flow meter (model DPM3, Kofloc) connected to compressed gas cylinders through a gas manifold. Nitrogen and O_2_ gas flow was adjusted to maintain 16% or 26% O_2_ by monitoring the O_2_ concentration in the downstream arm of the mass flowmeter with an O_2_ analyzer (LC-240UW, Vise Medical) when horses ran under hyperoxia and hypoxia.

### Blood and muscle sampling

Before leading a horse onto the treadmill, an 18-gauge catheter (Surflow, Terumo, Tokyo, Japan) was inserted into the carotid artery under local anesthesia (2% xylocaine), and an 8-F introducer (MO95H-8, Baxter International, Deerfield, IL, USA) was placed in the jugular vein. A Swan–Ganz catheter (SP5107U, Becton Dickinson, Franklin Lakes, NJ, USA) was then advanced through the jugular vein, with its tip positioned in the pulmonary artery. Correct placement was confirmed by measuring pressure at the catheter tip using a pressure transducer (P23XL, Becton Dickinson). Arterial blood samples and mixed venous blood samples in the pulmonary artery were drawn into heparinized syringes during the final 40 s of each exercise. The samples were stored on ice until analysis immediately following the experiment. Samples were analyzed using a blood gas analyzer (ABL800 FLEX, Radiometer, Copenhagen, Denmark), and O_2_ saturation and O_2_ concentrations were measured using a hemoximeter (ABL80 FLEX-CO-OX, Radiometer) set to its equine algorithm. The pulmonary artery temperature during exercise was measured at each blood sampling using a thermistor of the Swan–Ganz catheter connected to a cardiac output computer (COM-2; Baxter International) and was used to correct the blood gas measurements. After measuring blood gases and oximetry, blood samples were centrifuged (AX-511, Tomy Industrial, Tokyo, Japan) at 1740 ***g*** for 10 min to measure plasma lactate concentrations using a lactate analyzer (Biosen S-Line, EKF-diagnostic GmbH, Barleben, Germany).

In the present study, the gluteus medius muscle was selected for analyses because of its well-characterized metabolic profile and extensive use in previous studies examining exercise adaptation. (Bryan et al., 2017; Eivers et al., 2010, 2012; McGivney et al., 2009; Takahashi et al., 2024, 2025). The muscle biopsy sampling site was set at one-third of the distance from the coxal tuber on an imaginary line drawn from the coxal tuber to the root of the tail. The skin was locally anesthetized by subcutaneous injection of 0.5 ml of 2% lidocaine (Sandoz K. K., Tokyo, Japan). Given that sampling depth has been shown to affect muscle fiber-type composition (Rivero et al., 1993), but high reproducibility has been demonstrated when samples are taken from the same depth within a 5 cm radius (Wood et al., 1988), muscle samples were obtained from the same area (each sampling point was 2 cm apart) at a 5-cm depth of the middle of the gluteal muscle using a 13 gauge×3.9 cm coaxial introducer needle to accurately define depth and a 14 gauge×9 cm biopsy needle (SuperCore Biopsy instrument, Argon Medical Devices, Plano, TX, USA) before (pre), immediately after (post) and 4 h after each exercise trials. All muscle samples were frozen immediately in liquid nitrogen and stored at −80°C until analysis.

### Muscle glycogen assay

Muscle specimens (approximately 50 mg) were dissolved at 100°C in 30% KOH buffer saturated with Na_2_SO_4_. The homogenate was put on ice for 30 min after adding 99% ethanol. After glycogen in the solution was precipitated by centrifugation at 10,000 ***g*** for 10 min at 4°C, glycogen pellets were hydrolyzed to glucose in 1 mol l^−1^ HCl at 100°C for 2 h. After neutralization with 1 mol l^−1^ NaOH, glucose concentrations in the solution were determined using a kit (Glucose CII kit, Fujifilm Wako, Osaka, Japan) according to the manufacturer's instructions. Glycogen concentrations are expressed as glucosyl units per gram wet mass.

### Metabolomics

Metabolomic measurements were carried out at Human Metabolome Technologies, Inc. (Tsuruoka, Japan). All metabolomics samples were processed and analyzed within the same batch. The metabolites were analyzed using capillary electrophoresis (CE)-time of flight (TOF) mass spectrometry (MS) (Agilent CE-TOFMS system) and CE-triple quadrupole MS (CE-QqQMS) (Agilent CE and 6460 Triple Quad LC/MS systems, Agilent Technologies, Santa Clara, CA, USA). Cationic and anionic metabolites were analyzed using a fused-silica capillary (50 µm i.d.×80 cm) with cation buffer solution (p/n: H3301-1001; Human Metabolome Technologies) and anionic buffer solution (p/n: H3302-1023; Human Metabolome Technologies), respectively, as the electrolyte. CE-TOFMS and CE-QqQMS data were analyzed using automatic integration software (MasterHand v.2.17.1.11, Keio University, Japan) and MassHunter (Agilent Technologies), respectively.

### RNA extraction and sequencing

Total RNA was extracted from muscle tissues using the RNeasy Plus Universal Mini Kit (cat. no. 73404, Qiagen, Venlo, Limburg, The Netherlands) in the QIAcube Connect System (cat. no. 9002864, Qiagen). The samples were examined for integrity using the Agilent RNA 6000 Nano Kit (cat. no. 5067-1511; Agilent Technologies) with a bioanalyzer (Agilent Technologies). After evaluating the RNA integrity number, the RNA was used for library preparation for mRNA-seq. The mRNA-seq analysis was conducted at the Department of Sports Medicine, Open Facility Network Office, University of Tsukuba (Tsukuba, Ibaraki, Japan). Libraries were created using the NEBNext Ultra II RNA Library Prep Kit for Illumina and the NEBNext Poly(A) mRNA Magnetic Isolation Module (cat nos. E7770 and E7490, New England Biolabs, Ipswich, MA, USA), according to the manufacturer's instructions. The concentration and size distribution of the libraries were measured using the Agilent DNA 7500 kit (cat. no. 5067-1506, Agilent Technologies) with a bioanalyzer. The libraries were sequenced using NextSeq 500/550 v2.5 (75 cycles) kits (cat. no. 20024906, Illumina, San Diego, CA, USA) on the NextSeq 500 System (Illumina). The sequencing was performed with paired-end reads of 36 bases. The obtained FASTQ files were exported, and the basic information of the run data was checked using CLC Genomics Workbench 23.0.2 software (Qiagen).

### Bioinformatic and statistical analyses

All data were analyzed using MetaboAnalyst software (v.5.0, https://www.metaboanalyst.ca/), integrated differential expression and pathway (iDEP) (http://bioinformatics.sdstate.edu/idep96), and GraphPad Prism software (v10.1.1, Macintosh, GraphPad Software, La Jolla, CA, USA). To analyze the pre- and post-exercise data across the three oxygen conditions, a two-way ANOVA (exercise×O_2_) was performed, followed by a Tukey–Kramer multiple comparison test to detect differences within each oxygen condition and between pre- and post-exercise values. A one-way ANOVA was performed to analyze metabolite data detected as a result of the metabolomic analysis. Using the calculated *P*-value of the one-way ANOVA, the adjusted *P*-value [false discovery rate (FDR)] was calculated according to the method of Benjamini and Hochberg. After the FDR cut-off, Tukey's HSD test was used to determine the differences among groups. Regarding RNA-seq data, raw read counts from each sample were normalized using the EdgeR algorithm (minimal counts per million=0.5, pseudo count=4, gene median). The top 2000 most variable genes were clustered into four groups using the *k*-means algorithm. Enrichment *P*-values were calculated on the basis of the one-sided hypergeometric test, which was then adjusted for multiple testing using the Benjamini–Hochberg procedure. Based on the terms in the gene ontology biological process (GOBP) and Kyoto Encyclopedia of Genes and Genomes (KEGG), gene set enrichment analysis was performed using horse (*Equus caballus*)-specific gene annotation databases were used to ensure accurate functional interpretation. Differentially expressed genes were detected using DEseq2 (FDR cut-off=0.05, minimal fold change=1.5). Statistical significance was set as *P*<0.05.

## RESULTS

### Cardiometabolic responses

**Fig. 2.**
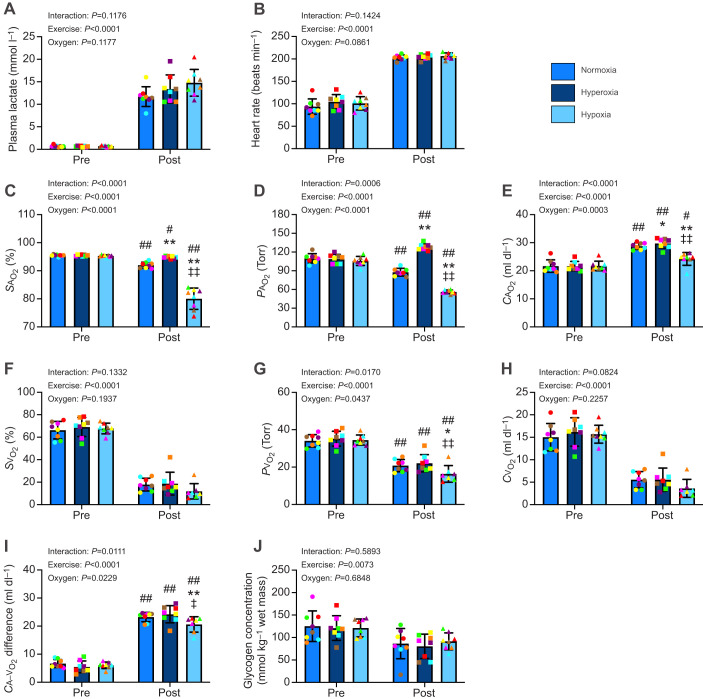
**Cardiometabolic responses to exercise.** (A) Plasma lactate concentration and (B) heart rate before and after exercise with *F*i_O_2__ of 0.21 (normoxia), 0.26 (hyperoxia) and 0.16 (hypoxia). (C,F) O_2_ saturation, (D,G) O_2_ partial pressure and (E,H) O_2_ content (E,H) in the arterial (C–E) and mixed-venous (F–H) blood. (I) The arterial–mixed-venous O_2_ difference and (J) glycogen concentration in the gluteus medius muscle. Data are presented as means±s.d. with individual values. Two-way ANOVA (exercise×O_2_), followed by Tukey–Kramer's multiple comparison test, was used to detect differences within the same O_2_ condition, and between pre- and post-exercise. ^##^*P*<0.01, ^#^*P*<0.05: significantly different from pre-exercise within the same condition. **P<0.01, *P<0.05: significantly different from normoxia at the given timepoint. ^‡‡^*P*<0.01, ^‡^*P*<0.05: significantly different from hyperoxia at the given timepoint.

We initially evaluated whether different *F*i_O_2__ levels effectively alter arterial O_2_ availability and assessed the potential effects on other cardiometabolic parameters. Before exercise (i.e. before inhaling hyperoxic or hypoxic gas), there were no significant differences in any measured cardiometabolic parameters between the gas mixes (Fig. 2). Exercise significantly increased plasma lactate concentrations (*P*<0.0001; Fig. 2A) and heart rate (*P*<0.0001; Fig. 2B) compared with pre-exercise, without any significant differences between the three O_2_ conditions. Arterial O_2_ saturation (*S*a_O_2__) was significantly decreased post-exercise compared with pre-exercise (*P*<0.0001), regardless of the O_2_ condition (Fig. 2C). Arterial O_2_ partial pressure (*P*a_O_2__) was decreased after exercise compared with pre-exercise under normoxia (*P*=0.0012) and hypoxia (*P*<0.0001), but was increased under hyperoxia (*P*=0.0010) (Fig. 2D). Arterial O_2_ content (*C*a_O_2__) was significantly increased post-exercise compared with pre-exercise in all O_2_ environments (*P*<0.05; Fig. 2E). Post-exercise values of *S*a_O_2__, *P*a_O_2__ and *C*a_O_2__ were significantly higher with hyperoxia (*P*<0.05) and lower with hypoxia (*P*<0.01) compared with normoxia (Fig. 2C–E). Exercise significantly decreased venous O_2_ saturation (*S*v_O_2__) (*P*<0.0001), with no difference among the three O_2_ conditions (Fig. 2F). Venous O_2_ partial pressure (*P*v_O_2__) was decreased following exercise compared with before exercise, and *P*v_O_2_ _values were significantly lower under hypoxia than under normoxia (*P*<0.05) and hyperoxia (*P*<0.01) (Fig. 2G). Additionally, exercise significantly decreased venous O_2_ content (*C*v_O_2__) (*P*<0.0001), with no difference between the O_2_ conditions (Fig. 2H). The *C*a–v_O_2__ difference was increased post-exercise compared with pre-exercise, but it was attenuated under hypoxia compared with normoxia (*P*=0.0088) and hyperoxia (*P*=0.0277) (Fig. 2I). Exercise significantly decreased muscle glycogen concentrations compared with pre-exercise (*P*=0.0073), with no significant effect of O_2_ conditions (Fig. 2J).

### Metabolomic analysis

**Fig. 3.**
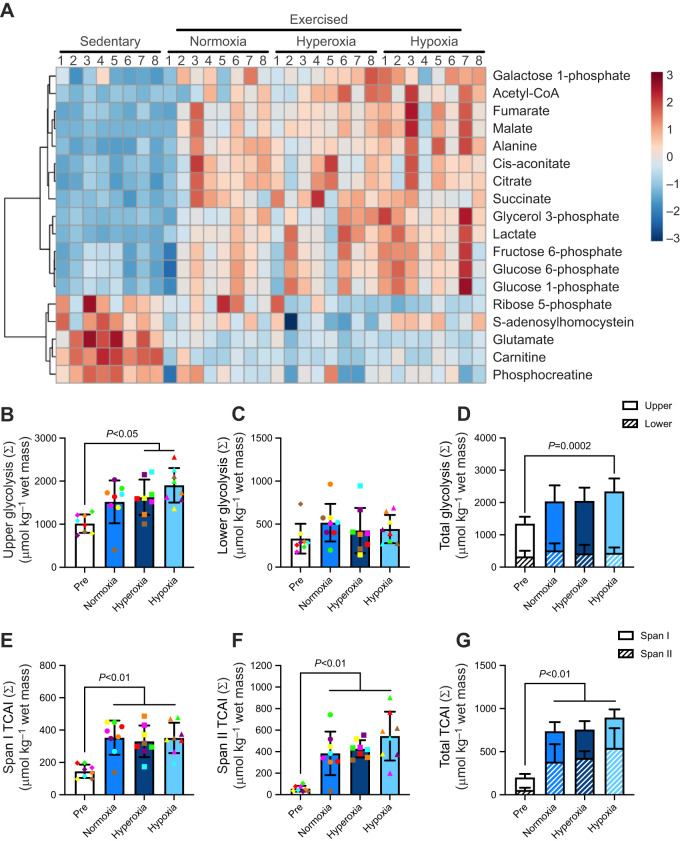
**Significant features of metabolomics, and glycolytic and TCA intermediates.** (A) A heatmap showing hierarchical clustering of significant features. (B–D) The sum of upper (B), lower (C) and total (D) intermediates in the glycolytic pathway. (E–G) The sum of span I (E), span II (F) and total (G) intermediates in the tricarboxylic acid (TCA) cycle. Data are presented as means±s.d. with individual values, and were analyzed using integrated differential expression and pathway (MetaboAnalyst). For metabolite data detected as a result of metabolomic analysis, one-way ANOVA followed by false discovery rate (FDR) cut-off using the Benjamini–Hochberg method was performed to identify significant features. After the FDR cut-off, Tukey's HSD test was used to determine the differences among groups.

To obtain deeper insights into intermediary metabolism associated with muscle bioenergetics, we conducted a metabolomic analysis focusing on central energy pathways, such as glycolysis, the tricarboxylic acid cycle (TCA) cycle, amino acid metabolism and nucleic acid metabolism. This analysis detected 96 metabolites (Table S1), of which 18 were identified as significant features as shown by one-way ANOVA, followed by the FDR cut-off (<0.05) (Fig. 3A; Fig. S1, Table S2). Among them, a significant difference between the O_2_ conditions was only observed for S-adenosylhomocysteine concentrations, which were significantly lower after hyperoxic exercise than after hypoxic exercise (*P*<0.05) (Fig. S1, Table S2). Regarding the remaining significant features, including phosphocreatine, glycolytic metabolites and TCA intermediates and anaplerotic substrates, we observed exercise-induced changes, but no significant differences were found between the three O_2_ conditions (Fig. S1, Table S2).

To further investigate intermediary metabolism, we calculated the total metabolite content within the glycolytic pathway and the TCA cycle. Although the total content of upper glycolytic intermediates (Σ upper glycolysis; i.e. the sum of glucose 6-phosphate, fructose 6-phosphate, glucose 1-phosphate, fructose 1,6-disphosphate, dihydroxyacetone phosphate and glyceraldehyde 3-phosphate) was increased after hyperoxic (*P*=0.0339) and hypoxic (*P*=0.0023) exercise, but not after normoxic exercise (*P*=0.0760) compared with pre-exercise (Fig. 3B), no significant changes were observed in lower glycolytic intermediates (Σ lower glycolysis; i.e. the sum of 2,3-diphosphoglycerate, 2-phosphoglyceric acid, 3-phosphoglycerate phosphoenolpyruvate and pyruvate) (Fig. 3C). Additionally, the total content of glycolytic intermediates (Σ upper glycolysis+Σ lower glycolysis) was significantly increased only after hypoxic exercise compared with pre-exercise (*P*=0.0002); however, no significant differences in these changes were observed among the three O_2_ conditions (Fig. 3D). Exercise significantly increased TCA intermediates of span I (Σ span I TCAI; i.e. citrate, cis-aconitase, isocitrate and 2-oxoglutarate) and span II (Σ span II TCAI; succinyl-CoA, succinate, malate, fumarate and oxaloacetate), and the total TCA intermediates (Σ span I+Σ span II) compared with pre-exercise (*P*<0.01), with no significant differences between the O_2_ conditions (Fig. 3E–G). Overall, the metabolic responses to acute exercise did not vary, irrespective of the *F*i_O_2__ gas used in this study.

### RNA-seq

**Fig. 4.**
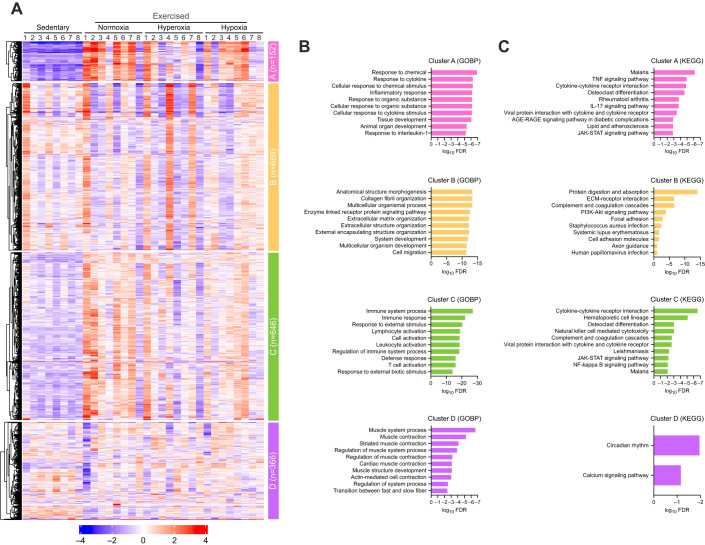
**Gene clustering and gene set enrichment analysis.** (A) A heatmap of the top 2000 most variable genes separated into four clusters using *k*-means algorithm. (B,C) Gene set enrichment analysis based on (B) gene ontology biological process terms (GOBP) and (C) Kyoto Encyclopedia of Genes and Genomes (KEGG). Enrichment *P*-values are calculated based on one-sided the hypergeometric test, which was then adjusted for multiple testing using the Benjamini–Hochberg procedure and converted to FDR. The significant pathways are sorted by FDR, and only the top 10 pathways are shown. Data were analyzed using integrated differential expression and pathway (iDEP).

To gain an overview of transcriptional activity following acute exercise with different *F*i_O_2__ levels, we performed RNA-seq using a muscle biopsy performed 4 h after acute exercise. We separated the top 2000 genes into four clusters using the non-hierarchical *k*-means clustering (Fig. 4A; Table S3). Fig. 4B shows the top 10 pathways of the gene set enrichment analysis based on GOBP terms of each cluster. The main annotations of each cluster are as follows: (A) response to chemicals and cytokines, (B) extracellular and structure organization, (C) immune and leukocyte responses and (D) muscle contraction and development. Additionally, the top 10 enriched genes based on KEGG are shown in Fig. 4C. Our data suggest that acute exercise, regardless of the O_2_ environment, induces distinct gene expression patterns compared with no exercise.

**Fig. 5.**
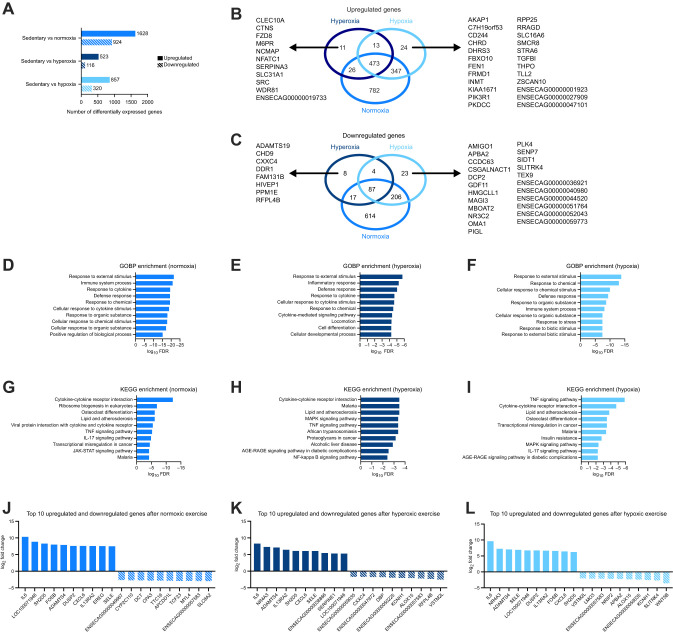
**Exercise-responsive differentially expressed genes and gene sets.** (A) The number of differentially expressed genes (DEGs; FDR=0.05, minimal fold change=1.5) and (B,C) Venn diagrams of upregulated (B) and downregulated (C) genes. (D–I) Gene set enrichment analysis based on (D–F) gene ontology biological process terms (GOBP) and (G–I) Kyoto Encyclopedia of Genes and Genomes (KEGG). (J–L) The top 10 upregulated and downregulated genes following exercise with *F*i_O_2__ of (J) 0.21 (normoxia), (K) 0.26 (hyperoxia) and (L) 0.16 (hypoxia). Enrichment *P*-values are calculated based on one-sided the hypergeometric test, which is then adjusted for multiple testing using the Benjamini–Hochberg procedure and converted to FDR. The significant pathways are sorted by FDR. Data were analyzed using integrated differential expression and pathway (iDEP).

To elucidate the gene responses to hyperoxic and hypoxic exercise, we analyzed differentially expressed genes. Among the three O_2_ conditions, exercise under normoxia induced the highest number of gene responses (upregulated: 1628; downregulated: 924) (Table S4), followed by hypoxia (upregulated: 857; downregulated: 320) (Table S6), whereas hyperoxia induced the fewest (upregulated: 523; downregulated: 116) (Table S5; Fig. 5A). A Venn diagram shows the number of upregulated (Fig. 5B) or downregulated (Fig. 5C) genes across the three O_2_ environments, and the genes were unique to each O_2_ condition. Specifically, hyperoxic exercise uniquely upregulated 11 genes and downregulated 8 genes, whereas hypoxic exercise uniquely upregulated 24 genes and downregulated 23 genes (Table S7). Additionally, the top 10 upregulated genes based on GOBP and KEGG of each cluster are shown in Fig. 4D–I and Tables S8–S10. Among them, three GOBP terms (defense response, response to an external stimulus, and response to chemicals) and four KEGG terms (cytokine–cytokine receptor interaction, lipid and atherosclerosis, malaria, and TNF signaling pathway) were shared across the O_2_ conditions. Furthermore, Fig. 5J–L shows the top 10 upregulated and downregulated genes following exercise under each O_2_ condition. Among them, five genes were common across the three O_2_ conditions. The fold-change and FDR values for each exercise condition of the top 20 DEGs are presented in Table S11. Taken together, these findings suggest that the overall gene response remains similar across O_2_ environments but is attenuated with changes in inspired O_2_ concentrations.

## DISCUSSION

### Overview of the present findings

Exercise training under hyperoxic and hypoxic environments has become popular for athletic and therapeutic purposes (Cardinale and Ekblom, 2018; Millet et al., 2016). However, metabolic and transcriptional responses and their relationships, particularly in horses, have not been fully determined. The present study showed that inspiring different *F*i_O_2__ levels was effective to change arterial O_2_ availability during exercise. Although the hypoxic condition reduced the *C*a–v_O_2__ difference compared with the normoxic and hyperoxic conditions, neither the metabolic nor transcriptional response showed distinct changes across the O_2_ conditions. These observations suggest that the metabolic and transcriptional responses induced by short period of high-intensity exercise are not dependent on the availability of O_2_ in skeletal muscle of Thoroughbred horses.

### Metabolic responses

#### Arterial O_2_ availability during exercise

Based on observations in humans, the activation of mitochondrial oxidative phosphorylation at the onset of exercise is believed to follow an exponential time course, such that the rate of ATP hydrolysis initially exceeds the rate of ATP production from oxidative phosphorylation (Bangsbo et al., 2000; Wilson et al., 1994). This transient shortfall in oxidative ATP supply is compensated by substrate-level phosphorylation, including ATP production through phosphocreatine hydrolysis and glycolysis. One possible mechanism contributing to this lag in oxidative phosphorylation during the onset of exercise is that a metabolic inertia, including lags in enzyme activation or substrate availability, requires a certain amount of time to produce the reducing equivalents required to drive the electron transport chain as shown using canine muscle (Grassi et al., 1998a,b, 2023, 2011). Another possibility based on human findings is that a suboptimal O_2_ supply limits the production of ATP in the mitochondria of some muscle fibers (Hughson and Kowalchuk, 1995; Tschakovsky and Hughson, 1999; Whipp and Ward, 1990). The amount of O_2_ delivered to the mitochondria is determined by convective O_2_ delivery, including CaO_2_ and blood flow, and diffusive O_2_ delivery, which is affected by the *P*_O_2__ gradient from red blood cells to mitochondria (Wagner, 1996). In the present study, post-exercise *S*a_O_2__, *C*a_O_2__ and *P*a_O_2__ were higher in the hyperoxic condition and lower in the hypoxic condition than in the normoxic condition. In Thoroughbred horses, breathing with varied *F*i_O_2__ levels did not change cardiac output during exercise (Wagner et al., 1996), suggesting a similar blood flow across O_2_ conditions. These data suggest that convective and diffusive O_2_ delivery are increased by hyperoxia and reduced by hypoxia.

It is worth noting that previous studies have shown that convective O_2_ delivery and peripheral O_2_ diffusion are not limiting factors for *V*_O_2__ kinetics during transitions from rest to 60% of *V*_O_2__ peak in the isolated dog gastrocnemius muscle (Grassi et al., 1998a,b). However, in a subsequent study, this was unlikely to be the case during transitions from rest to 100% of *V*_O_2__ peak (Grassi et al., 2000). Therefore, we cannot exclude the possibility that exercise at a higher intensity than in the present study (95% *V̇*_O_2_,max_) may elicit different responses under hyperoxia.

#### Substrate-level phosphorylation during exercise

The lack of sparing of substrate-level phosphorylation during exercise with the hyperoxic condition in this study is consistent with previous human studies using enzymatic spectrophotometric assays, which showed no effect of hyperoxia during the initial 1.5–2 min of exercise at 65% and 90% peak O_2_ uptake (Evans et al., 2001; Savasi et al., 2002). There are other studies in humans showing a reduction in substrate-level phosphorylation during exhaustive exercise lasting 4–5 min while breathing room air at 1.4 atmospheric pressure (Linnarsson et al., 1974) or 60% O_2_ (Linossier et al., 2000). The difference in these results may be explained by the lack of time course measurements. Indeed, it is reported that hyperoxia did not affect substrate-level phosphorylation during the initial minute of exercise, but reduced muscle glycogenolysis and lactate production during 15 to 40 min of steady-state exercise in humans (Stellingwerff et al., 2005, 2006). Using ^31^P-magnetic resonance spectroscopy, another human study showed that breathing 10%, 21% and 100% O_2_ similarly increased phosphocreatine hydrolysis during the initial phase of exercise, whereas breathing 10% and 21%, but not 100%, further decreased phosphocreatine levels in the subsequent phase (Haseler et al., 2004). Collectively, these findings suggest that oxidative phosphorylation at the onset of exercise is not limited by O_2_ availability in the current experimental settings.

In humans, during moderate-intensity exercise (50–65% *V̇*_O_2_,max_), hypoxia induces greater glycogenolysis and lactate accumulation than with normoxia (Brooks et al., 1998; Green et al., 1992; Parolin et al., 2000). However, the present study showed no significant differences in muscle glycogen content, glycolytic intermediates, lactate levels or plasma lactate concentrations after a 2-min exercise bout at 95% *V̇*_O_2_,max_ in the different O_2_ conditions in horses. Supporting our observations, a previous human study reported no difference in muscle glycogen, muscle lactate or peak blood lactate concentrations during 4 to 5 min of maximal exercise while breathing room air at 0.68 and 1.00 atmospheric pressure (Linnarsson et al., 1974). Additionally, exercise at the same relative workload (85% *V̇*_O_2_,max_), but at a lower absolute power, during hypoxia resulted in a glycogen use similar to that observed during normoxia in humans (Young et al., 1982). Taken together, these studies and our study suggest that hypoxemia does not necessarily augment glycogenolysis and lactate production during high-intensity exercise.

#### Oxidative metabolism during exercise

Several human studies have reported that whole-body O_2_ consumption and O_2_ consumption across the exercising leg are preserved during submaximal exercise under hypoxic conditions (Adams and Welch, 1980; Knight et al., 1993; Parolin et al., 2000). In contrast, the present study showed that the post-exercise *C*a–v_O_2__ difference was significantly diminished in the hypoxic condition compared with the normoxic and hyperoxic conditions in Thoroughbreds. Other human studies have shown that leg O_2_ consumption during near-maximal and maximal exercise, but not low-intensity exercise, was reduced in hypoxia compared with normoxia (Richardson et al., 1995, 1998). These data suggest that hypoxia impedes increasing muscle O_2_ extraction from blood during exercise in an intensity-dependent manner. The decline in O_2_ consumption and the *C*a–v_O_2__ difference could be explained by a reduction in the driving gradient from blood to tissue in the hypoxic environment (Richardson et al., 1995). Importantly, several lines of evidence have shown that intracellular *P*_O_2__ during exercise or muscle contraction with normoxia and even hypoxia does not drop to a critical level (Molé et al., 1999; Richardson et al., 1998; Takakura et al., 2010), at which mitochondrial respiration is compromised (0.1–0.5 Torr, where 1 Torr=133.322 Pa) (Chance and Quistorff, 1977). During muscle contraction under hindlimb perfusion, myoglobin O_2_ saturation decreases to a greater extent with perfusate at low O_2_ fractions in rats (Takakura et al., 2017). Collectively, these findings indicate that a greater use of myoglobin-associated O_2_ reserve in the hypoxic environment may compensate for the decline in muscle O_2_ extraction from blood, resulting in no significant difference in substrate-level phosphorylation during exercise in different O_2_ conditions.

### Transcriptomic responses

#### Common gene response to exercise

To the best of our knowledge, this is the first study to examine transcriptomic responses to exercise under varying O_2_ conditions, particularly following hyperoxic exercise. Although we found that numerous genes, including well-known exercise-responsive genes, such as *NR4A3*, *PPARGC1A*, *PDK4* and *VEGFA* (Takahashi et al., 2024), were altered following exercise irrespective of the O_2_ environment, a reduced number of differentially expressed genes was observed under hyperoxia and hypoxia compared with normoxia. This finding suggests that neither hyperoxic nor hypoxic environments enhance transcriptomic changes generally associated with skeletal muscle adaptation to exercise in Thoroughbreds.

#### Gene response unique to hyperoxic/hypoxic exercise

Although several human studies have reported the potential of hyperoxic training for improving exercise performance (Cardinale and Ekblom, 2018; Kon et al., 2019, 2020; Perry et al., 2005), the underlying mechanisms remain unclear. The lack of mechanistic insights into skeletal muscle adaptation is possibly because measurements were limited to specific targets of interest in isolation. Here, using RNA-seq analysis, we identified several genes that were unique to the O_2_ conditions. Among the 11 genes uniquely upregulated following hyperoxic exercise, we found that two – *mannose-6-phosphate receptor* (*M6PR*) and *cystinosin* (*CTNS*) – are associated with lysosomal storage diseases. *M6PR* plays a critical role in protein trafficking, specifically directing lysosomal enzymes to lysosomes. In Pompe disease, a lysosomal glycogen storage disorder, enzyme replacement therapy (ERT) with recombinant acid α-glucosidase (GAA) has been developed; however, in the mouse model of this disease, the therapeutic response in skeletal muscle is notably attenuated compared with that in cardiac muscle (Raben et al., 2003). This diminished efficacy has been attributed to the relatively low abundance of M6PR in skeletal muscle (Raben et al., 2005). Pharmacological upregulation of *M6PR* via clenbuterol administration has been shown to enhance the effectiveness of ERT by improving receptor-mediated uptake of GAA into mouse muscle tissue (Koeberl et al., 2011). Moreover, the *CTNS* gene encodes cystinosin, a lysosomal membrane transporter responsible for exporting cystine. Mutations in *CTNS* lead to cystinosis, a rare autosomal recessive lysosomal storage disorder characterized by the pathological accumulation of cystine in various organs and tissues. In mouse models, *CTNS* deficiency has been associated with reduced muscle fiber cross-sectional area and muscle strength, likely owing to impaired myogenesis, activation of proteolytic pathways and elevated expression of pro-inflammatory cytokines (Cheung et al., 2016). Other investigations have shown that hyperoxia can lead to increased numbers of lysosomes, altered lysosomal structure, and potentially increased activity of lysosomal enzymes in cultured neuroblastoma cells and isolated perfused rat liver (Abraham et al., 1968; Zheng et al., 2011). These findings suggest that the upregulation of *M6PR* and *CTNS* following hyperoxic exercise may represent an adaptive mechanism to preserve muscle integrity by enhancing lysosomal function. We also observed that NFATC1, a key regulator of muscle fiber type switching in response to exercise (Ehlers et al., 2014), was upregulated exclusively following hyperoxic exercise. In contrast, Ppm1E, which encodes an AMPK phosphatase (Voss et al., 2011), was downregulated only after hyperoxic exercise, suggesting that hyperoxia may modulate AMPK-related metabolic pathways.

The beneficial effects of hypoxic training are primarily driven by hypoxia inducible factor-1α (HIF-1α) (Lundby et al., 2009). In this study, we observed that *HIF-1α* gene expression was upregulated in response to normoxic and hypoxic exercise but not to hyperoxic exercise in equine skeletal muscle. Although HIF-1α is degraded by prolyl hydroxylase domain (PHD) enzymes under conditions of sufficient intracellular O_2_ availability, HIF-1α is stabilized in a hypoxic environment and forms a dimer with HIF-1β, enabling its role in gene regulation (Semenza, 2009). Thus, HIF-1α is considered to be regulated post-transcriptionally, with few studies showing changes in transcription (Wenger et al., 1997). In the present study, although we did not observe significant differences in *HIF-1α* gene expression among the three oxygen conditions, PIK3R1, a known target of HIF-1α, was upregulated only in response to hypoxic exercise. Additionally, hypoxic exercise selectively upregulated *thrombopoietin* (*THPO*) and *a-kinase anchoring protein 1* (*AKAP1*), both of which have been implicated in cellular protection against hypoxia (Baker et al., 2015; Marin, 2020). We previously reported that treatment with a chemical that facilitates Hif-1α protein accumulation by inhibiting PHD enzymes resulted in increased glycolytic enzyme activity in mouse skeletal muscle (Abe et al., 2015), and that hypoxic training improved endurance performance to a greater extent than normoxic training in Thoroughbred horses (Mukai et al., 2021; Wang et al., 2020). Together, it is possible that HIF-1α mediates adaptive responses through its post-transcriptional regulation, but not through its transcriptional activity.

In addition to genes known to be respond to hypoxia, there was also evidence of changes in the expression of several genes that regulate muscle adaptations following only hypoxic exercise, such as *AKAP1* and *FBXO10*, which are required for maintenance of mitochondrial integrity (Bhat et al., 2024; Hoffman et al., 2015; Merrill and Strack, 2014), as well as *DHRS3* and *GDF11*, which are related to muscle regeneration (Egerman et al., 2015; Jacques et al., 2023). Interestingly, expression of *DCP2*, which is required for RNA degradation, was downregulated by prolonged intermittent hypoxia in mouse skeletal muscle (Attaway et al., 2023). Our results suggest that, despite the lower number of genes that were altered by exercise under hyperoxic or hypoxic conditions compared with normoxic conditions, the potential for a specific response to each O_2_ condition cannot be ruled out.

### Additional considerations and future perspectives

In the present study, we used omics approaches, such as metabolomics and RNA-seq. Although metabolomics is a comprehensive approach that enabled us to determine substrate concentration in the tissue, it does not reflect metabolic flux *in vivo*. Additionally, gene expression levels represent only the initial step toward protein synthesis. Therefore, RNA-seq is unable to clarify regulatory mechanisms occurring at post-transcriptional and translational levels. Moreover, we collected muscle samples 4 h after exercise, as previous studies have shown that the 4-h post-exercise time point exhibits the highest number of differentially expressed genes and the greatest magnitude of transcriptional changes in equine skeletal muscle (Bryan et al., 2017; Eivers et al., 2010, 2012; McGivney et al., 2009, 2010). Therefore, we consider this time point to be the most appropriate for our study. However, future research examining multiple time points may be necessary to fully elucidate the time course of gene expression changes in response to different oxygen environments, as shown in a human study, not all genes show the similar patterns of regulation (Bishop et al., 2023). Furthermore, the relatively small sample size (*n*=8) and inherent inter-individual variability – particularly relevant in transcriptomic analyses – should be acknowledged as potential limitations. These factors may influence the robustness of the detected gene expression patterns and should be taken into consideration when extrapolating our findings to broader equine populations.

Another consideration is the intensity of exercise, which is a critical factor to consider when interpreting our findings. In the present study, the exercise protocol across all conditions was identical and performed at a high intensity (95% of *V̇*_O_2_,max_ measured under normoxia). This uniform intensity likely elicited substantial metabolic and transcriptional responses independent of the O_2_ environment. In practical settings, power output at a particular heart rate and *V̇*_O_2_,max_ typically increase under hyperoxic conditions and decrease under hypoxic conditions. Notably, an improvement in performance has been observed following hyperoxic training when exercise is conducted at the same relative intensity in humans (Perry et al., 2005). These data suggest that performance improvements observed with hyperoxic training may be attributed to the increased total power output. Future studies should investigate the metabolic and transcriptional responses to exercise performed at the same relative intensity under varying O_2_ conditions, which could provide deeper insights into the skeletal muscle adaptations relevant to practical applications.

It would be worth noting unique traits of Thoroughbreds. Although arterial O_2_ levels during exercise increased with hyperoxia and decreased with hypoxia compared with normoxia, they increased in all conditions compared with pre-exercise levels. This finding is different to that in humans, in which arterial O_2_ content drops during exercise, especially when inhaling a hypoxic gas mixture (Dempsey and Wagner, 1999). This unique observation in horses is due to the ability to release red blood cells from the spleen during exercise (Hanzawa et al., 1995), thereby increasing the total hemoglobin mass (Fig. S2), hematocrit and thus arterial O_2_ levels (Wagneft et al., 1995). These observations lead to the question of whether the current findings are also applicable to humans.

In addition to the muscle-specific responses examined here, systemic physiological markers may provide additional insights when evaluating the effects of altered oxygen availability. In horses, responses of cytokines, such as interleukin-1 receptor agonist (IL-1ra), IL-10 and IL-13, to exercise were shown to be different depending on training status (Plisak et al., 2023; Witkowska-Piłaszewicz et al., 2020). Moreover, previous studies have shown that acute exercise increased the circulating factors, such as microRNA and extracellular vesicles, related to processes of muscle remodeling, immunity and inflammation in horses (Cappelli et al., 2018; Milczek-Haduch et al., 2025). Furthermore, circulating factors such as serum amyloid A and the testosterone/cortisol ratio have been reported to change with exercise intensity and training load in horses (Grzędzicka et al., 2023). Although evidence suggests that exercise-induced secretion and cargo content of extracellular vesicles are influenced by oxidative stress (Witkowska-Piłaszewicz et al., 2024), at present the effects of O_2_ condition during exercise are unknown. Given that exercise under hypoxic conditions elicited a more pronounced natural killer cell response in humans compared with exercise in normoxia (Klokker et al., 1993), further study using cell-type-specific analysis is required. Taken together, incorporating these systemic markers in future studies would provide a more comprehensive understanding of the interplay between local muscle responses and systemic regulation under hypoxic and hyperoxic exercise conditions.

## Conclusions

In this study, we showed that using different *F*i_O_2__ levels was effective in changing O_2_ availability in the arterial blood of Thoroughbred horses during a 2-min exercise bout performed at 95% *V̇*_O_2_,max_. However, biochemical and metabolomic analyses showed little, if any, effects of O_2_ conditions on muscle bioenergetics. *In silico* analysis of RNA-seq data showed that numerous genes, including well-known exercise-responsive genes, such as *NR4A3*, *PPARGC1A*, *PDK4* and *VEGFA*, were altered following exercise irrespective of the O_2_ environment, although the total number of significantly altered genes was reduced under both hyperoxia and hypoxia compared with normoxia. Notably, genes related to lysosomal activity, such as *M6PR* and *CTNS*, were upregulated only after hyperoxic exercise, whereas hypoxia-responsive genes beyond the canonical HIF-1α response, including *PIK3R1*, *THPO* and *AKAP1*, were upregulated by hypoxic exercise. The present data suggest that changing arterial O_2_ availability does not necessarily affect the metabolic response to exercise or subsequent overall transcriptional patterns in skeletal muscle. However, the identification of specific genes that responded uniquely to either hyperoxic or hypoxic exercise implies the potential for distinctive physiological adaptations under each condition. Our findings, combined with insights from recent multi-omics studies, are also potentially valuable for establishing biomarkers of fitness, training adaptation and overtraining in equine sports medicine.

## Supplementary Material

10.1242/jexbio.250956_sup1 Supplementary information

Table S1. Metabolomics data

Table S2. Significant features

Table S3. Gene sets enrichment of each cluster

Table S4. Genes up- or down-regulated by exercise with normoxia

Table S5. Genes up- or down-regulated by exercise with hyperoxia

Table S6. Genes up- or down-regulated by exercise with hypoxia

Table S7. Genes up- or down-regulated specific to hyperoxic or hypoxic exercise

Table S8. Gene sets altered after normoxic exercise

Table S9. Gene sets altered after hyperoxic exercise

Table S10. Gene sets altered after hypoxic exercise

Table S11. Top 20 up- or down-regulated genes after exercise
