# Supplementary material for: Effects of hypoxia and hyperoxia on exercise-induced metabolomic and transcriptomic profiles in equine skeletal muscle
Source: J Exp Biol. 2025 Dec 17;228(24):jeb250956. doi: 10.1242/jeb.250956 (PMC12752499; doi:10.1242/jeb.250956)
Supplement: Supplementary information [file jexbio-228-250956-s1.pdf]

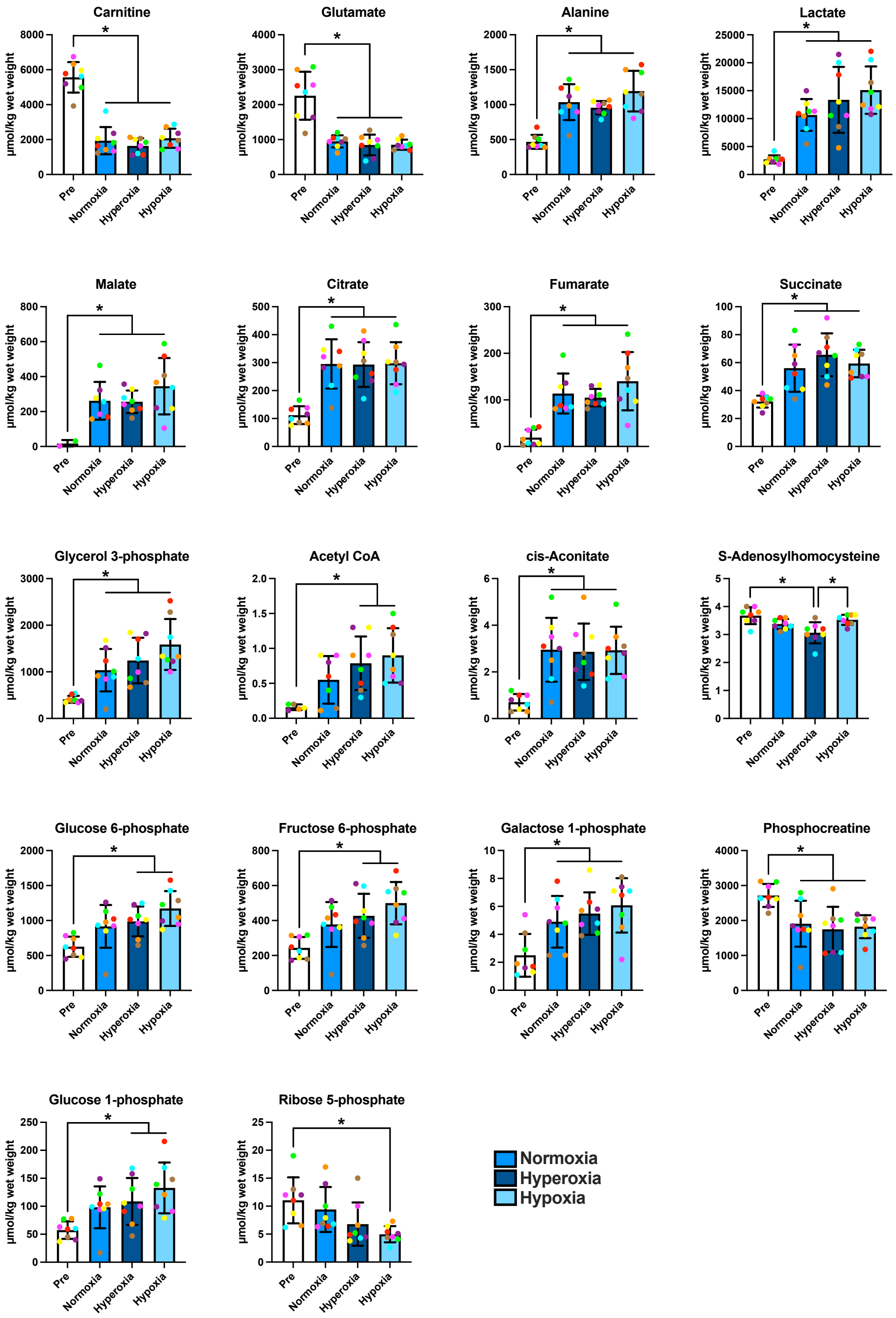


**Fig. S1. Significant features of metabolomics**

Data are presented as means ± SD with individual value. For metabolite data detected as a result of metabolomic analysis, one-way ANOVA followed by false discovery rate (FDR) cutoff using the method of Benjamini and Hochberg was performed to identify significant features. After the FDR cutoff, Tukey's HSD test was used to determine the differences among groups. *denotes significant difference at the level of $p<0.05$.

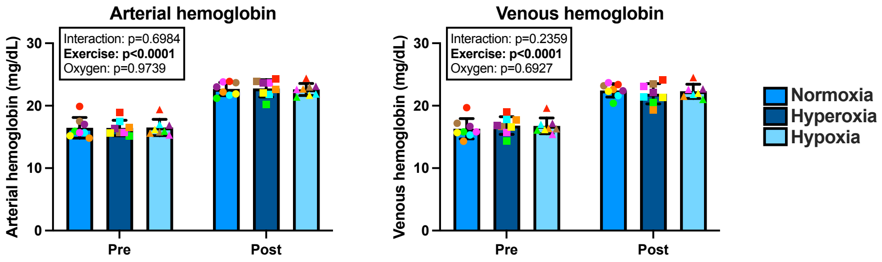


**Fig. S2. Hemoglobin concentration in the blood**

Hemoglobin concentration in the arterial and mixed-venous blood. Data are presented as means ± SD with individual value. Two-way analysis of variance (ANOVA; exercise × $O_2$) was performed to detect an interaction and main effects.

**Table S1. Metabolomics data**

Available for download at
https://journals.biologists.com/jeb/article-lookup/doi/10.1242/jeb.250956#supplementary-data

**Table S2. Significant features**

Available for download at
https://journals.biologists.com/jeb/article-lookup/doi/10.1242/jeb.250956#supplementary-data

**Table S3. Gene sets enrichment of each cluster**

Available for download at
https://journals.biologists.com/jeb/article-lookup/doi/10.1242/jeb.250956#supplementary-data

**Table S4. Genes up- or down-regulated by exercise with normoxia**

Available for download at
https://journals.biologists.com/jeb/article-lookup/doi/10.1242/jeb.250956#supplementary-data

Journal of Experimental Biology • Supplementary information

**Table S5. Genes up- or down-regulated by exercise with hyperoxia**

Available for download at
https://journals.biologists.com/jeb/article-lookup/doi/10.1242/jeb.250956#supplementary-data

**Table S6. Genes up- or down-regulated by exercise with hypoxia**

Available for download at
https://journals.biologists.com/jeb/article-lookup/doi/10.1242/jeb.250956#supplementary-data

**Table S7. Genes up- or down-regulated specific to hyperoxic or hypoxic exercise**

Available for download at
https://journals.biologists.com/jeb/article-lookup/doi/10.1242/jeb.250956#supplementary-data

**Table S8. Gene sets altered after normoxic exercise**

Available for download at
https://journals.biologists.com/jeb/article-lookup/doi/10.1242/jeb.250956#supplementary-data

**Table S9. Gene sets altered after hyperoxic exercise**

Available for download at
https://journals.biologists.com/jeb/article-lookup/doi/10.1242/jeb.250956#supplementary-data

**Table S10. Gene sets altered after hypoxic exercise**

Available for download at
https://journals.biologists.com/jeb/article-lookup/doi/10.1242/jeb.250956#supplementary-data

**Table S11. Top 20 up- or down-regulated genes after exercise**

Available for download at
https://journals.biologists.com/jeb/article-lookup/doi/10.1242/jeb.250956#supplementary-data
